# Exosomal miR-183-5p promotes angiogenesis in colorectal cancer by regulation of FOXO1

**DOI:** 10.18632/aging.103145

**Published:** 2020-05-03

**Authors:** Anquan Shang, Xuan Wang, Chenzheng Gu, Wenfang Liu, Junjun Sun, Bingjie Zeng, Chen Chen, Ping Ji, Junlu Wu, Wenqiang Quan, Yiwen Yao, Weiwei Wang, Zujun Sun, Dong Li

**Affiliations:** 1 Department of Laboratory Medicine, Shanghai Tongji Hospital, Tongji University School of Medicine, Shanghai, P.R. China; 2 Department of Pharmacy, Putuo People’s Hospital, Shanghai, P.R. China; 3 Department of General Surgery, Shanghai Tongji Hospital, Tongji University School of Medicine, Shanghai, P.R. China; 4 Department of Internal Medicine V-Pulmonology, Allergology, Respiratory Intensive Care Medicine, Saarland University Hospital, Homburg, Germany; 5 Department of Pathology, The Sixth People's Hospital of Yancheng, Yancheng, P.R. China

**Keywords:** colorectal cancer, exosome, microRNA-183-5p, FOXO1, angiogenesis

## Abstract

Exosomes play important roles in proliferation and microenvironment modulation of many types of cancers, including colorectal cancer (CRC). However, the inhibitory effect of CRC cells-derived exosomes in angiogenesis has not been fully discussed. In this study, the roles of microRNA-183-5p (miR-183-5p) in abundant in exosomes secreted from the CRC cells were investigated. Initially, microarray analysis was employed to determine the differentially expressed miRNAs. Exosomes isolated from CRC cells were co-cultured with HMEC-1 cells to explore the role of exosomes in angiogenesis. Further, the effects of CRC cell-derived exosomal miR-183-5p on proliferation, invasion and tube formation abilities of HMEC-1 cells were assessed. The preventative effect of exosomal miR-183-5p *in vivo* was measured in nude mice. Initially, it was found that FOXO1 was downregulated while miR-183-5p was upregulated in CRC. Additionally, the inhibition of miR-183-5p was suggested to suppress proliferation, invasion and tube formation abilities of HMEC-1 cells through upregulating FOXO1. Then, *in vitro* assays demonstrated that CRC cell-derived exosomes overexpressing miR-183-5p contributed to an enhanced proliferation, invasion and tube formation abilities of HMEC-1 cells. Furthermore, *in vivo* experiments confirmed the tumor-promotive effects of CRC cell-derived exosomal miR-183-5p. Collectively, our study demonstrates that the CRC cell-derived exosomes overexpressing miR-183-5p aggravates CRC through the regulation of FOXO1. Exosomes overexpressing miR-183-5p might be a potential treatment biomarker for CRC.

## INTRODUCTION

Colorectal cancer (CRC) ranks as the third most common malignancy, with its mortality just second to lung cancer. In 2018, CRC had 1.8 million new cases and caused 881,000 deaths [1]. Despite the continuous advancement in clinical practice and screening technologies, CRC diagnostic efficiency remain below expectation in most countries, including the developed world [2]. In recent years, epigenetic alterations, highlighting their functional roles in the progression of CRC have been highlighted in a plethora of researches, and the insight into the role and mechanism of highly effective molecules could lend support for development of prognostic biomarker and novel therapeutic regimes [3]. Besides, Angiogenesis is a hallmark process towards tumor growth and metastasis [4]. However, it was assumed that the development of more efficient anti-angiogenic therapy may only possess modest value in the second-line and subsequent-line therapy for CRC [5], which entails approaches to concurrently or successively target the tumor microenvironment [6].

The role of microRNAs (miRNAs or miRs) have been well established in various types of cancers, either as tumor suppressors or oncomiRs. miRNA-based therapy has been proposed as a promising preclinical application [7, 8]. For instance, miR-23a was shown to induce the transition from indolent to invasive CRC, indicating its role as a facilitator in the progression of cancer [9]. An integrated analysis of CRC-related microRNA datasets revealed that hsa-miR-183-5p was up-regulated in CRC [10] while miR-183-5p has also been implicated in other types of cancers. High expression levels of miR-183-5p displayed correlation with low rates of overall survival, predictive of worse prognoses of patients with renal cell cancer [11]. Furthermore, miR-183-5p was shown to play an oncogenic role in lung adenocarcinoma via its interaction with various target genes [12]. Intriguingly, miR-183 enhances the survival of non-small cell lung cancer cells by targeting forkhead box O1 (FOXO1) [13]. As one of FOXO isoforms, FOXO1 is a crucial target of insulin signaling involved in the regulation of metabolic homeostasis along with organismal survival at different levels, suggesting its strong association to angiogenesis and tumor development [14]. Hence, it is attractive to identify whether miR-183-5p could enhance the progression CRC through targeting FOXO1.

Exosomes represent small nanovesicles derived from all types of cells in the body, particularly tumor cells, which are capable of regulating intercellular communications [15]. Tumor-derived exosomes harbor a different array of cargoes that possess the ability to accelerate angiogenesis, which ultimately modulates cancer invasiveness [16]. A recent study placed emphasis on the critical roles of exosomal miRNAs in cancer progression that could induce angiogenesis and accelerate metastasis [17]. Based on these findings, the main objective of the study was to identify the potential roles of exosomal miR-183-5p in CRC, and further elucidate the underlying molecular mechanisms. Hence, it was hypothesized that CRC cells-derived exosomes enhance the angiogenesis of microvascular endothelial cells through the delivery of miR-183-5p, which may provide further insight into novel targets for CRC treatment.

## RESULTS

### Exosomes secreted from CRC cells exhibit high miR-183-5p expression

**Figure 1:**
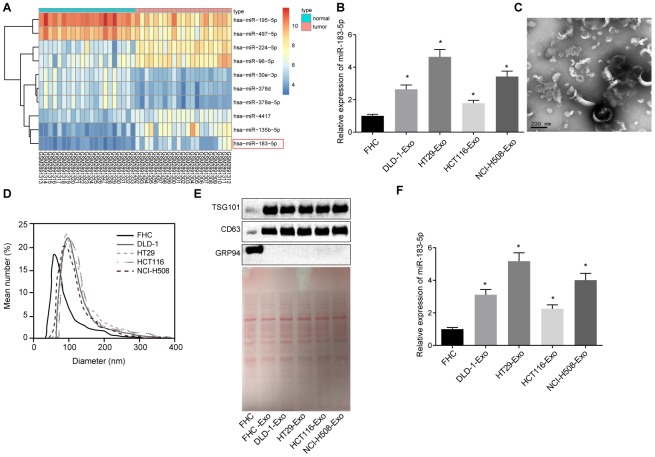
**Exosomes secreted from CRC cells exhibit high expression of miR-183-5p.** (**A**) heatmap of differentially expressed miRNA screened from GSE108153, with the horizontal coordinate representative of the sample number; the longitudinal coordinate reflective of the differentially expressed miRNA; the color gradation histogram on the right indicative the level of gene expression where each box in the diagram indicates the expression level of a gene in a sample. The warmer color reflected the higher expression, and the colder color reflected the lower expression; (**B**) expression of miR-183-5p in CRC cell lines detected via RT-qPCR; (**C**) identification of structure of exosomes by transmission electron microscopy; (**D**) analysis of size distribution of exosomes was conducted by NanoSight NS300 Nanoparticle Tracking Analyzer; (**E**) protein bands and expression of TSG101, CD63 and GRP94 as detected by Western blot analysis; (**F**) expression of miR-183-5p in exosomes secreted from CRC cells detected via RT-qPCR; * *p* < 0.05 compared with the FHC cell. Measurement data were expressed as mean ± standard deviation; comparisons among multiple groups were assessed by one-way analysis of variance. Cell experiment was repeated three times.

R language was employed for the differential analyses of the CRC-related microarray data GSE108153. A total of 49 differentially expressed miRNAs were screened out from GSE108153 followed by the construction a heatmap illustrating the expression of the first 10 miRNAs (Figure 1A). Our results identified miR-183 as one of the differentially expressed miRNAs, whose expression in CRC tissues was significantly higher than that of normal tissues. Previous studies have highlighted the carcinogenic effects of miR-183 in various cancers such as synovial sarcoma, rhabdomyosarcoma and colon cancer [18]. Hence, we intended to investigate the functions of miR-183 from a CRC point of view. Next, the expression of miR-183-5p in different CRC cell lines was determined by means of RT-qPCR, the results of which indicated an increase in the expression of miR-183-5p in the DLD-1, HT29H, CT116 and NCI-H508 cells when compared to the FHC cells (p < 0.05) (Figure 1B). The exosomes extracted from the CRC cells were analyzed under a transmission electron microscope. The exosomes were identified to be solid and compact, with a typical bilayer membrane structure, presenting a disc or cup shape. The average diameter of exosomes was approximately 60 nm (Figure 1C). Meanwhile, the size and distribution of exosomes assessed by the NanoSight NS300 Nanoparticle Tracking Analyzer (Figure 1D), verified that the diameters of most of the microparticles were in the range of exosomes (30-150 nm). Western blot analysis revealed higher TSG101 and CD63 expression, accompanied by lower expression of GRP94 in the DLD-1-, HT29-, CT116- and NCI-H508-derived exosomes when compared to the FHC cells (p < 0.01), indicative of successful exosome extraction (Figure 1E). The expression of miR-183-5p in the extracted exosomes was determined by RT-qPCR. In comparison to the FHC cell-derived exosomes, DLD-1-, HT29-, CT116- and NCI-H508-derived exosomes exhibited higher expression levels of miR-183-5p (p < 0.05) (Figure 1F). The HT29 cells displayed relatively high expression levels of miR-183-5p, and were subsequently selected for further experimentation.

### HT29 cell-derived exosomes promote proliferation, migration and tube formation abilities of HMEC-1 cells through overexpressing miR-183-5p

HT29-Exos were co-cultured with HMEC-1 cells for 48 h to elucidate the role of HT29-Exo in CRC. The uptake of red fluorescence PKH-26 labeled exosome by HMEC-1 cells was examined under an inverted fluorescence microscope after the HMEC-1 cells had been co-cultured with HT29-Exo (Supplementary Figure 1A). Based on RT-qPCR results, increased miR-183-5p expression was observed in the HMEC-1 cells co-cultured with HT29-Exo (Supplementary Figure 1B). To ascertain whether the HT29 cells and HMEC-1 cells exerted their effects through exosomes, we co-cultured HT29 cells pretreated with exosome inhibitors, with HMEC-1 cells, followed by the addition of co-cultured HT29 cells and HMEC-1 cells. Evaluation of proliferation, migration and the tube formation abilities of the HMEC-1 cells were subsequently assessed. Our results revealed that co-culture with HT29 cells led to enhanced proliferation, migration and tube formation abilities of the HMEC-1 cells (p < 0.05). After pre-treatment with 5 μM GW4869 (an inhibitor of exosome exocytosis; HY-19363, MCE, USA) on HT29 cells, exosome exocytosis was inhibited, along with suppressed proliferation, migration and tube formation abilities of HMEC-1 cells (p < 0.05, Supplementary Figure 1C–1E). The results suggested that HT29-Exos could promote proliferation, migration and tube formation abilities of HMEC-1 cells.

**Figure 2:**
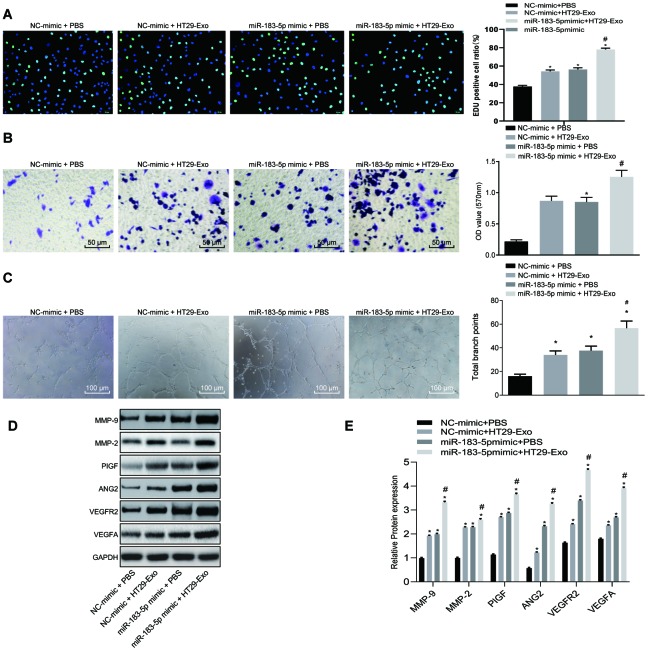
**HT29 cell-derived exosomes overexpressing miR-183-5p promote proliferation, migration tube formation abilities and angiogenesis of HMEC-1 cells.** (**A**) EdU assay was applied to detect the proliferation of the HMEC-1 cells following treatment with HT29-Exo and miR-183-5p mimic (Scale bar = 50 μm); (**B**) HMEC-1 cell migration was detected by Transwell assay after treatment of HT29-Exo and miR-183-5p mimic (Scale bar = 50 μm); (**C**) tube formation abilities of HMEC-1 cell were detected by tube formation assay after treatment of HT29-Exo and miR-183-5p mimic (Scale bar = 100 μm); (**D**–**E**) expression of angiogenesis-related proteins (VEGFA, VEGFAR2, ANG2, PIGF, MMP-2 and MMP-9) in HMEC-1 cells after treatment of HT29-Exo and miR-183-5p mimic was also detected by western blot analysis; * *p* < 0.05 compared with the NC-mimic + PBS group, # *p* < 0.05 compared with the NC-mimic + HT29-Exo, or miR-183-5p mimic + PBS groups. Measurement data were presented as mean ± standard deviation; comparisons among multiple groups were assessed by one-way analysis of variance. Cell experiment was repeated three times.

To elucidate the mechanism by which HT29-Exo promotes the proliferation, migration and in vitro tube formation abilities of HMEC-1 cells, HT29-Exos were co-cultured with HMEC-1 cells with or without overexpressed miR-183-5p. The results revealed that co-culture with HT29-Exo alone or combined with overexpressed miR-183-5p could led to an increased number of EdU positive cells, and promotion of the migration and tube formation abilities in HMEC-1 cells (p < 0.05). Both HMEC-1 cells overexpressing miR-183-5p and those co-culture with HT29-Exo demonstrated even more significant increase (p < 0.05, Figure 2A–2C). Moreover, Co-cultured with HT29 Exo, HMEC-1 cells displayed an up-regulated expression of VEGFA, VEGFAR2, ANG2, PIGF, MMP-2 and MMP-9 (p < 0.05), which has even more significant increase in co-cultured cells overexpressing miR-183-5p (p < 0.05, Figure 2D–2E). Additionally, the inhibition of miR-183-5p was found to effectively reverse the stimulatory effects associated with HT29-Exo on the facilitation of proliferation, migration and tube formation of HMEC-1 cells, as well as the increase in the expression of angiogenesis-related proteins (p < 0.05, Supplementary Figure 2). Hence, based on these results, we concluded that HT29 cell-derived exosomes promoted the proliferation, migration and tube formation abilities of HMEC-1 cells through the overexpression of miR-183-5p.

### HT29 cell-derived exosomes overexpressing miR-183-5p promote proliferation, migration and tube formation of HMEC-1 cells through inhibiting FOXO1

A total of 4,256 differentially differentiated genes were screened out through the differential expression analyses of the microarray data GSE89076. The target genes of miR-183-5p were predicted based on the miRDB, mirDIP, TargetScan, miRTarBase, and RNA22, with 376, 904, 502, 353, and 5,208 genes identified respectively from the five aforementioned databases. The predicted results from these five databases were intersected with the differentially expressed genes screened from GSE89076, with this data visualized on a Venn map (Supplementary Figure 3A). FOXO1 was identified as an intersecting gene regulated by miR-183 based on its differential expression. The expression of FOXO1 in GSE89076 is illustrated in Supplementary Figure 3B. The expression of FOXO1 in CRC tissues was found to be lower than normal tissues. In addition, low expression level of FOXO1 in CRC was indicated by the UALCAN database (Supplementary Figure 3C). Previous researches implicated the low expression of FOXO1 in CRC [19], suggesting the potential of FOXO1 as an inhibitor of tumor angiogenesis [20, 21]. However, the molecular mechanisms are yet to be sufficiently depicted. Multiple lines of evidence showed that miR-183-5p can be secreted into the extracellular system in the form of exosome [22]. We subsequently asserted the notion that CRC cell-derived exosomes expressing miR-183-5p could influence angiogenesis by regulating FOXO1. Next, miRDB was employed to predict the binding site of miR-183-5p on FOXO1, and rendered that the site 236-242 on FOXO1 3'UTR has high complementarity with miR-183-5p (Supplementary Figure 3D). A dual-luciferase reporter assay was performed to verify whether FOXO1 was indeed the target of miR-183-5p. The results indicated that miR-183-5p mimics reduced the luciferase activity of pGL-FOXO1 WT (p < 0. 05), while this was not observed in the luciferase activity of pGL-FOXO1 MUT (p > 0 05), indicating that miR-183-5p could bind to and downregulate the expression of FOXO1 (Supplementary Figure 3E). In order to further verify that FOXO1 was the target gene of miR-183-5p, the expression of FOXO1 in the cells overexpressing miR-183-5p was detected using RT-qPCR and Western blot analysis. The results indicated that overexpression of miR-183-5p could reduce the expression of FOXO1 in HMEC-1 cells (Supplementary Figure 3F–3H). These results confirmed FOXO1 as a potential target gene of miR-183-5p.

**Figure 3:**
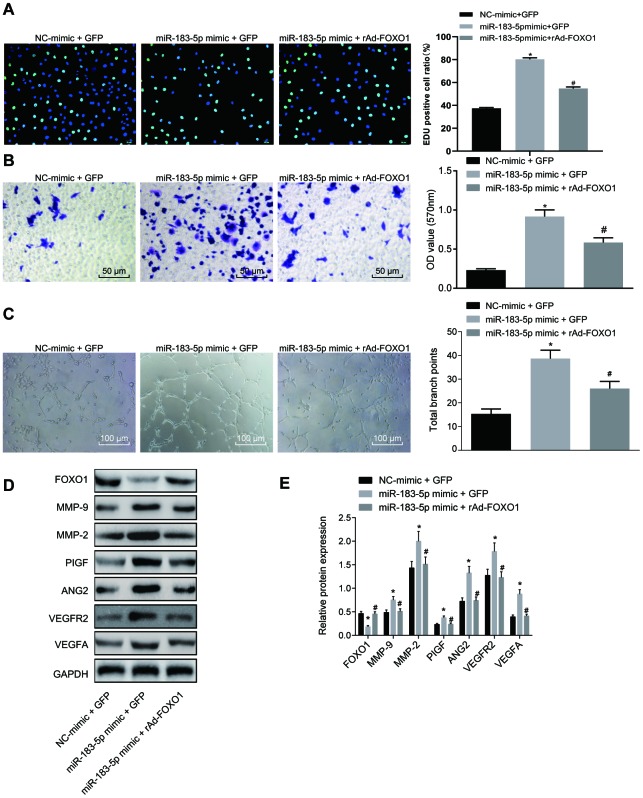
**Overexpression of miR-183-5p promotes proliferation, migration and tube formation abilities of HMEC-1 cells by targeting FOXO1.** (**A**) EdU assay was applied to detect proliferation of HMEC-1 cells after treatment of miR-183-5p mimic and rAd-FOXO1 (Scale bar = 50 μm); (**B**) HMEC-1 cell migration was detected by Transwell assay after treatment of miR-183-5p mimic and rAd-FOXO1 (Scale bar = 50 μm); (**C**) tube formation abilities of HMEC-1 cell were detected by tube formation assay after treatment of miR-183-5p mimic and rAd-FOXO1(Scale bar = 100 μm); (**D**–**E**) expression of angiogenesis-related proteins (VEGFA, VEGFAR2, ANG2, PIGF, MMP-2 and MMP-9) and FOXO1 in HMEC-1 cells after treatment of miR-183-5p mimic and rAd-FOXO1 was also detected by western blot analysis; * *p* < 0.05 compared with the NC-mimic + GFP group, # *p* < 0.05 compared with the miR-183-5p mimic + GFP group; Measurement data were presented as mean ± standard deviation; comparisons among multiple groups were assessed by one-way analysis of variance. Cell experiment was repeated three times.

**Figure 4:**
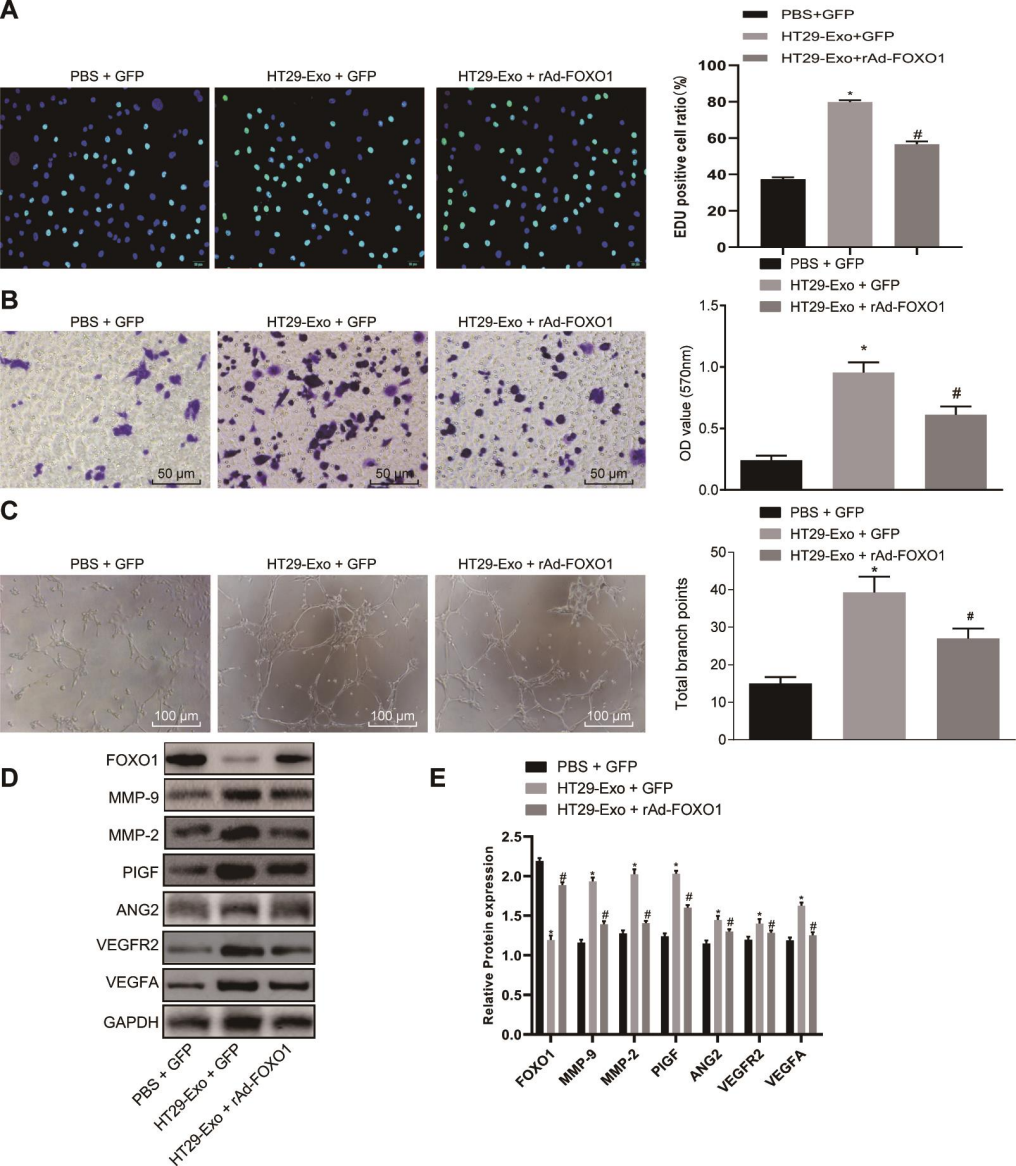
**HT29 cell-derived exosomes promote proliferation, migration and tube formation abilities of HMEC-1 cells by inhibiting FOXO1.** (**A**) EdU assay was applied to detect proliferation of HMEC-1 cells after treatment of HT29-Exo and rAd-FOXO1 (Scale bar = 50 μm); (**B**) HMEC-1 cell migration was detected by Transwell assay after treatment of HT29-Exo and rAd-FOXO1 (Scale bar = 50 μm); (**C**) tube formation abilities of HMEC-1 cell were detected by tube formation assay after treatment of HT29-Exo and rAd-FOXO1 (Scale bar = 100 μm); (**D**–**E**) expression of angiogenesis-related proteins (VEGFA, VEGFAR2, ANG2, PIGF, MMP-2 and MMP-9) and FOXO1 in HMEC-1 cells after treatment of HT29-Exo and rAd-FOXO1 was detected by western blot analysis; *p < 0.05 compared with the PBS + GFP group, #*p* < 0.05 compared with the HT29-Exo + GFP group; Measurement data were presented as mean ± standard deviation; comparisons among multiple groups were assessed by one-way analysis of variance. Cell experiment was repeated three times.

As FOXO1 is a target gene of miR-183-5p and exhibits low expression in CRC, we sought to determine whether FOXO1 was involved in the regulatory role of HT29-Exo in HMEC-1 cells. Our results revealed that overexpression of FOXO1 could reverse the effects of HT29-Exo or miR-183-5p overexpression on proliferation, migration and tube formation abilities of HMEC-1 cells (p < 0.05, Figures 3A–3C, 4A–4C). Subsequently, expression of angiogenesis-related proteins and FOXO1 in HMEC-1 cells was detected by Western blot analysis, demonstrating that treatment of HT29-Exo or overexpression of miR-183-5p could inhibit the expression of FOXO1 while increasing expression of angiogenesis-related proteins (p < 0.05), which could be reversed by the overexpression of FOXO1 (p < 0.05, Figures 3D–3E, 4D–4E). These evidences verified that HT29 cell-derived exosomes overexpressing miR-183-5p promote the proliferation, migration and tube formation abilities of HMEC-1 cells by targeting FOXO1.

### Inhibition of miR-183-5p reduces the tumorigenic ability of HT29 cells and decreases the MVD in nude mice

**Figure 5:**
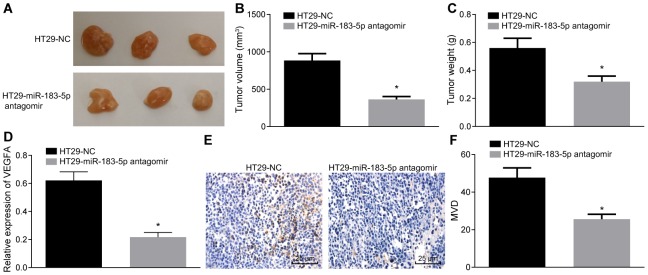
**Inhibition of miR-183-5p in HT29 cells reduces the tumorigenic ability and decreases the MVD in nude mice.** (**A**) tumorigenicity of HT29 cells in nude mice; (**B** and **C**) tumor volume and weight from nude mice; (**D**) serum level of VEGFA detected by ELISA; (**E**, **F**) immunohistochemical analysis (Scale bar = 25 μm) and quantitation of MVD in nude mice; *p < 0.05 compared with the HT29-NC group; Measurement data were presented as mean ± standard deviation; comparisons between two groups were conducted by means of independent *t*-test, n = 10.

Finally, the *in vitro* findings were subsequently confirmed in the *in vivo* experiments following the injection of HT29-miR-183-5p antagomir into nude mice. The HT29 cells treated with miR-183-5p antagomir were inoculated into nude mice. Three weeks after the injection, there was a hard tumor mass presenting as a distinct swelling of the upper abdomen of the nude mice. The nude mice were notably thin, anorexic, mentally depressed and exhibited stiffness in limb movement. The transplanted tumors were dissected, and were found to be ellipsoid or irregular in shape. All tumor tissues were confirmed by pathological examination. We found that inhibition of miR-183-5p could suppress tumorigenic ability of HT29 cells (Figure 5A), reduce tumor volume (Figure 5B) and tumor weight (Figure 5C) (all p < 0.05).

ELISA was then adopted to determine the serum VEGFA level, which showed that inhibition of miR-183-5p could decrease VEGFA level in serum (Figure 5D, p < 0.05). The MVD of nude mice was measured, the results demonstrated that the new vessels were a cell or cell cluster stained with brown color. In addition, the inhibition of miR-183-5p significantly decreased MVD in nude mice (Figure 5E, 5F, p < 0.05).

**Figure 6:**
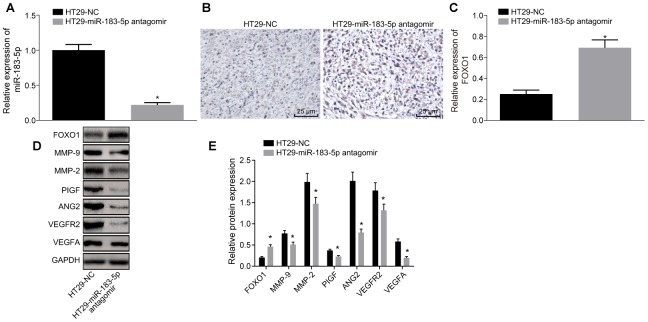
**Inhibition of miR-183-5p in HT29 cells reduces the expression of FOXO1 and angiogenesis-related factors in nude mice.** (**A**) expression of miR-183-5p in serum detected by RT-qPCR; (**B**, **C**) immunohistochemical detection of FOXO1 expression in tumor tissues of nude mice (Scale bar = 25 μm); (**D**–**E**) expression of angiogenesis-related proteins (VEGFA, VEGFAR2, ANG2, PIGF, MMP-2 and MMP-9) and FOXO1 in tumor tissues detected by Western blot analysis; *p < 0.05 compared with the HT29-NC group; Measurement data were presented as mean ± standard deviation; comparisons between two groups were conducted by means of independent *t*-test, n = 10.

The exosomes were extracted from serum, followed by detection of miR-183-5p expression by RT-qPCR. The results revealed that miR-183-5p antagomir decreased miR-183-5p expression in serum derived exosomes (Figure 6A, p < 0.05). Immunohistochemistry was employed to detect the expression of FOXO1, the results of which revealed that the expression of FOXO1 protein in mice injected with HT29 cells transfected with miR-183-5p antagomir was notably higher than that in the mice injected with HT29 cells transfected with NC (Figure 6B, 6C). Western blot analysis further indicated that inhibition of miR-183-5p led to a down-regulation in the expression of VEGFA, VEGFAR2, ANG2, PIGF, MMP-2 and MMP-9 along with up-regulated FOXO1 expression in tumor tissues (Figure 6D–6E, p < 0.05). These results suggested that inhibition of miR-183-5p as well as the overexpression of FOXO1 could diminish the tumorigenic ability of HT29 cells and decrease the MVD in nude mice.

## DISCUSSION

Exosomes possess the ability to transfer various types of cargos such as proteins, mRNAs, and miRNAs to recipient cells, which results in the activation and/or blockade of different cellular and molecular pathways to alter cell behaviors [23]. We performed this study to elucidate the role of exosomes derived from CRC cells containing miR-183-5p to mediate the angiogenesis of vascular endothelial cells. Both *in vitro* and *in vivo* assays provided evidences demonstrating that CRC cell-derived exosomes facilitate the proliferation, invasion and tube formation abilities of HMEC-1 cells by aberrantly higher level of miR-183-5p, which ultimately induced angiogenesis via downregulation of FOXO1.

A key initial finding of our study revealed that miR-183-5p was highly expressed in CRC cell-derived exosomes. Previous studies have suggested that miR-183-5p functions as an oncomiR inducing the proliferation, invasion and metastasis of malignancies such as pancreatic adenocarcinoma [24] as well as breast cancer [25]. Consistently, Chen et al. presented evidence demonstrating that the expression of miR-183-5p is markedly higher in CRC tissues than that of their normal counterparts [26]. Furthermore, the upregulation of miR-183-5p has been implicated in other types of cancers such as triple-negative breast cancer [27], as well as bladder cancer [28]. A previous study validated that the expression of miR-183-5p is notably higher in CRC tissues, consistent with the data from TCGA database [26]. A crucial finding of our study revealed that miR-183-5p was overexpressed in CRC cell-derived exosomes. CRC cell-derived exosomes carrying miR-183-5p confer stimulatory effects on proliferation, invasion as well as the tube formation abilities of HMEC-1 cells, highlighting the pro-angiogenic role of exosomal miR-183-5p. A previous study documented that an increase in the expression of miR-183 is closely correlated with advanced clinical stage, metastases and tumor progression of CRC, which was partially consistent with the findings of the present study [29]. We also showed that exosomal miR-183-5p stimulated angiogenesis, manifested by up-regulated expression of VEGFA, VEGFAR2, ANG2, PIGF, MMP-2 and MMP-9. It has been established that miRNAs exert functions on angiogenesis in CRC by mediating angiogenic factors VEGF, and ANG2. For instance, exosomal miR-25-3p derived from cancer cells stimulates vascular permeability and angiogenesis through modulating the expression of VEGFR2, ZO-1, occludin and Claudin5 in endothelial cells [30].

Furthermore, the present study demonstrated that exosomal miR-183-5p derived from CRC cells triggered a marked increase in the proliferation, migration and tube formation abilities of HMEC-1 cells by targeting FOXO1. Intriguingly, the overexpression of FOXO1 was observed to be capable of reversing the elevations in angiogenesis-related proteins induced by miR-183-5p. A previous study revealed that miR-183 displays oncogenic potential in CRC through the mediation of two oncogenes, EGR1 and PTEN [18]. Accumulating evidence has indicated that miR-183 binds to FOXO1 mRNA 3'UTR, and inhibits the translation of FOXO1 mRNA [13]. Studies have indicated that the human FOXO1 contains one functional miR-183 binding site which confers post-transcriptional regulation triggered by miR-183 [31]. FOXO1 has been speculated to be a significant mediator of vascular growth in addition to metabolic activity as well as the growth state of ECs by mechanistically inhibiting the signaling of c-MYC, an anabolic metabolism and growth mediator [32]. Also, the anti-angiogenic role of FOXO1 has been documented in gastric cancer, which when inhibited contributes to tumor growth by upregulating HIF-1α and VEGF [21]. The *in vivo* assays performed during our study provided further verification indicating that exosomal miR-183-5p suppresses the tumorigenic ability of CRC cells and tumor angiogenesis via the inhibition of FOXO1 expression, which was evidenced by a reduction in tumor volume and weight along with decreased serum VEGFA level and MVD.

Taken conjointly, our results supported the notion that miR-183-5p was first found to be highly expressed in CRC cell-derived exosomes, which triggers a marked increase in the proliferation, migration and tube formation abilities of HMEC-1 cells by targeting FOXO1 (Supplementary Figure 4). The overexpression of FOXO1 was observed to be capable of reversing the elevations in angiogenesis-related proteins induced by miR-183-5p. Our findings potentially shed new light on the theoretical underpinning of disease-specific miRNA delivery of exosomes as potential target in CRC diagnosis and treatment. However, the clinical application of exosomal miRNA at present is limited by the lack of effective exosome extraction technology.

## MATERIALS AND METHODS

### Ethical statement

This study was performed under the approval of the Animal Ethics Committee of Shanghai Tongji Hospital, Tongji University School of Medicine (2019tjdx217). Extensive efforts were made to ensure animal suffering was minimized.

### Microarray-based gene and miRNA analysis

CRC-related microarray data GSE108153 (miRNA) and GSE89076 (gene) from Gene Expression Omnibus (GEO) database (https://www.ncbi.nlm.nih.gov/geo/) (data available 2019-04-28) was employed for differential analyses. GSE108153 represents the miRNA expression data of 21 pairs of CRC tissues and normal tissues, and annotation platform is GPL19730 Agilent-046064 Unrestricted_Human_miRNA_V19.0_Microarray (Probe Name version). GSE89076 was representative of the expression data of 9 pairs of CRC tissues and normal tissues, which were applied for screening the differentially expressed genes, while the annotation platform GPL16699 Agilent-039494 SurePrint G3 Human GE v2 8x60K Microarray 039381 (Feature Number version). R language affy packet [33] was adopted to standardize the pretreatment of microarray data. In addition, the limma packet [34] was applied in order to identify the differentially expressed miRNA between CRC tissues and normal tissues. The corrected p value was referred to as adj.P.Val, the screening conditions for differentially expressed miRNA were as follows: |log2FoldChange(FC)|>1 and adj.P.Val<0.05, and the heatmap of differentially expressed miRNA was drawn. miRDB (http://www.mirdb.org/) (data available 2019-04-28), mirDIP (http://ophid.utoronto.ca/mirDIP/) (data available 2019-04-28), TargetScan (http://www.targetscan.org/vert_71/) (data available 2019-04-28), miRTarBase (http://mirtarbase.mbc.nctu.edu.tw/php/search.php) (data available 2019-04-28), and RNA22 (https://cm.jefferson.edu/rna22/) (data available 2019-04-28) were 5 miRNA-mRNA relationship prediction sites, which were deemed adequate for the prediction of the target genes of the differentially expressed miRNAs. Jvenn (http://jvenn.toulouse.inra.fr/app/example.html) (data available 2019-04-28) was applied to compare the differences between the target gene prediction results and GSE89076. The expression of the differentially expressed miRNAs was queried using the UALCAN database (http://ualcan.path.uab.edu/index.html) (data available 2019-04-28).

### Cell culture

Human colon epithelial cells (FHC), colon adenocarcinoma cell line (DLD-1), rectal adenocarcinoma cell line (HT29), colon cancer cell line (HCT116), and human microvascular endothelial cell line (HMEC-1) were purchased from ATCC, while the cecum adenocarcinoma cell line (NCI-H508) was purchased from the Center for basic Medical Cell, Institute of basic Medicine, Chinese Academy of Medical Sciences. The exosome-free fetal bovine serum (FBS) was produced by centrifugation (100,000 g) at 4°C overnight in order to ensure the removal of any bovine-derived exosomes [35]. The FHC cells were cultured in DMEM/F12 (Hyclone) medium containing 10% FBS. The DLD-1 cells were cultured in RPMI1640 medium containing 10% FBS, HT29 while the HCT116 cells were cultured in McCoy's 5A medium containing 10% FBS. The NCI-H508 cells were cultured in DMEM medium containing 10% FBS, and HMEC-1 cells were cultured in DMEM medium (31600-034, Hyclone, USA) comprised of 10% FBS (10099141, Gibco, USA) as well as streptomycin mixture. All cells were cultured at 37°C in a humidified atmosphere with 5% CO_2_. When cell confluency reached 90%, the cell passage procedure was performed accordingly.

### Extraction and characterization of exosomes

When the FHC, DLD-1, HT29, HCT116 and NCI-H508 cells reached 70-80% confluence, the complete culture medium was discarded. Cell culture medium (15-20 mL per cell line) was harvested to extract exosome in strict accordance with exosome extraction kit instructions (41201ES25, Shanghai Yeasen Company, Shanghai, China). The extracted exosomes were then stored at -80°C for subsequent experimentation.

The exosomes were identified under the guidance of a transmission electron microscope. Analysis of the exosome size distribution was performed using NanoSight NS300 (Nanosight, Marlvern, UK) [36]. The surface marker expression levels of the exosome including the exosome specific marker proteins tumor susceptibility gene 101 (TSG101), CD63, endoplasmic reticulum marker proteins heat shock protein 90, beta (Grp94), and member 1 (GRP94) were identified using Western blot analysis [37].

### RNA isolation and quantification

Total RNA was extracted as per the instructions provided by the TRIZOL kit (15596-018, Beijing Solarbio Technology Co., Ltd., Beijing, China). Reverse transcription was performed in accordance with the protocols of the miRNA reverse transcription kit (D1801, Harbin Haigene Testing co., Ltd., Harbin, China). An ABI7500 quantitative PCR instrument was employed for real-time fluorescence quantitative PCR. For normalization of sample-to-sample variation, Caenorhabditis elegans (cel)-miR-39 (Qiagen, Valencia, CA, USA) was employed as the housekeeping gene for serum samples. U6 was utilized as the housekeeping gene for cell samples while GAPDH was the housekeeping gene for FOXO1. The 2-ΔΔCt method was applied to calculate the relative expression of each target gene. The primers utilized were designed and synthesized by Takara Biotechnology Ltd., (Dalian, Liaoning, China) (listed in Table 1). The experiment was repeated 3 times independently.

**Table 1:** Primer sequences for RT-qPCR.

**Gene**	**Primer sequences**	
*miR-183-5p*	F: 5'-CGCGGTATGGCACTGGTAGA-3'	R: 5'-AGTGCAGGGTCCGAGGTATTC-3'
*FOXO1*	F: 5'-CAATGACCCCGCACGATTTC-3'	R: 5'-CATGGAGGGCGGATTGGAA-3'
*GAPDH*	F: 5'-GGCGTTCTCTTTGGAAAGGTGTTC-3'	R: 5'-GTACTCAGCGGCCAGCATCG-3'
*U6*	F: 5'-GCTTCGGCAGCACATATACTAAAAT-3'	R: 5'-CGCTTCACGAATTTGCGTGTCAT-3'
*cel-miR-39*	F: 5'-5'- ACACTCCAGCTGGGTCACCGGGTGTAAATC-3'	R: 5'-TGGTGTCGTGGAGTCG-3'

Note: RT-qPCR, reverse transcription quantitative polymerase chain reaction; F, forward; R, reverse; miR, microRNA; FOXO1, forkhead box O1; GAPDH, glyceraldehyde-3-phosphate dehydrogenase; cel, Caenorhabditis elegans.

### Western blot analysis

The total proteins were extracted from tissues, cells or exosomes using RIPA lysis buffer (R0010, Beijing Solarbio science and technology Co. ltd., Beijing, China) based on the instructions provided. The protein samples were separated by sodium dodecyl sulfate (SDS)-polyacrylamide gel electrophoresis (PAGE). The proteins were separated then transferred onto a polyvinylidene difluoride (PVDF) membrane. Membrane blockade was performed with 5% skimmed milk, followed by incubation with the primary antibody (all primary antibodies were rabbit antibody): TSG101 (ab30871, 1:1000, Abcam, Cambridge, UK), CD63 (ab68418, 1:1000, Abcam, Cambridge, UK), GRP94 (ab3674, 1:3000, Abcam, Cambridge, UK), CD31 (#3528, 1:1000, Cell Signaling Technology, Boston, MA, USA), fork head box O1 (FOXO1) (#2880, 1:500, Cell Signaling Technology, Boston, MA, USA), Vascular endothelial growth factor A (VEGFA) (ab46154, 1:1000, Abcam, Cambridge, UK, vascular endothelial growth factor receptor 2 (VEGFAR-2) (ab11939, 1:1000, Abcam, Cambridge, UK), angiopoietin 2 (ANG2) (ab8452, 1:500, Abcam, Cambridge, UK), phosphatidylinositol glycan anchor biosynthesis, class F (PIGF) (ab74778, 1:1000, Abcam, Cambridge, UK), matrix metallopeptidase 2 (MMP-2) (ab37150, 1:1000, Abcam, Cambridge, UK), matrix metallopeptidase 9 (MMP-9) (ab73734, 1:1000, Abcam, Cambridge, UK) and rat-anti GAPDH (ab8245, 1:5000, Abcam, Cambridge, UK). The membrane was incubated with HRP labeled goat anti-rabbit IgG (ab205718, 1:20000, Abcam, Cambridge, UK) or goat anti-rat (ab6789, 1:5000, Abcam, Cambridge, UK). The enhanced chemiluminescence (ECL) was adopted to visualize proteins, which were quantified using ImageJ 1.48u software (National Institutes of Health), with the ratio of gray value of each protein to the internal reference (GAPDH) obtained. Regarding the Western blot assay of the exosomal proteins, the PVDF membrane with transferred proteins was stained with Ponceau in order to ensure consistent protein loading between different lanes. The experiment was conducted in triplicate.

### Co-culture of PKH-26 labeled exosome with HMEC-1 cells

The extracted exosomes from the HT29 cells were treated according to the PKH-26 protocols (PKH26GL-1KT, Sigma-Aldrich, St. Louis, USA). The exosomes were incubated with PKH-26-Diluent C staining solution for 5 min. Next, 2 mL of 10% BSA supplemented with PBS (D8537) was then employed to terminate the staining process. Following the addition of sucrose solution (1.5 mL), exosomes were centrifuged at 190,000 g for 2 h under conditions of 2-8°C in order to collect the pellet, with the medium and interface layer aspirated off. The exosomal pellet was pipetted to obtain the suspension in PBS, which was then transferred to the Amicon filter column. Following the addition of 9 mL of PBS as well as 0.75 mL medium, the exosome suspension was centrifuged at 3000 g for 40 min to reduce the volume to 0.5-1 mL [37].

The HMEC-1 cells were then incubated with PKH-26 labeled exosomes for 24 h, fixed by 4% paraformaldehyde at room temperature for 30 min, and stained with DAPI (36308ES11, Yeasen Company, Shanghai, China) for 5 min. The cells were then analyzed and photographed under an inverted fluorescence microscope (DMi8, Leica, Wetzlar, Germany).

### Cell transfection

When the HMEC-1 cells reached 80%-90% confluence, the transfection process was conducted. The HMEC-1 cells were then treated with HT29 cells-secreted exosomes (HT29-exo) negative control (NC) and transfection of miR-183-5p mimic NC (PBS + NC-mimic group), HT29-Exo NC and transfection of miR-183-5p mimic (PBS + miR-183-5p mimic group), HT29-Exo and transfection of miR-183-5p mimic NC (HT29-Exo + NC-mimic group), HT29-Exo and transfection of miR-183-5p-mimic (HT29-Exo + miR-183-5p mimic group), HT29-Exo NC and transfection of miR-183-5p inhibitor NC (PBS + NC inhibitor group), HT29-Exo and transfection of miR-183-5p inhibitor NC (HT29-Exo + NC inhibitor group), HT29-Exo and transfection of miR-183-5p inhibitor (HT29-Exo + miR-183-5p inhibitor group), transfection of miR-183-5p mimic NC and infection of adenovirus-FOXO1 NC (NC mimic + green fluorescent protein [GFP] group), transfection of miR-183-5p mimic and infection of adenovirus-FOXO1 NC (miR-183-5p mimic + GFP group), transfection of miR-183-5p mimic and infection of adenovirus-FOXO1 (miR-183-5p mimic-rAd-FOXO1 group); HT29-Exo NC + infection of adenovirus-FOXO1 NC (PBS + GFP group), HT29-Exo and infection of adenovirus-FOXO1 NC (HT29-Exo + GFP group), HT29-Exo and infection of adenovirus-FOXO1 (HT29-Exo + rAd-FOXO1 group). The doses of miR-183-5p mimic and miR-183-5p inhibitor were both 50 nM. Transfection process of cells was conducted in accordance with the protocols of the Lipofectamine 2000 (11668-019, Invitrogen, New York, California, USA). FOXO1 adenovirus packaging was provided by Genomeditech co. ltd. (Shanghai, China). The HMEC-1 cells were transfected with 5 μl of FOXO1 adenovirus.

After transfection, the cells were cultured at 37°C in a humidified atmosphere with 5% CO_2_. After 6 h had elapsed, the original culture medium was replaced with DMEM containing 10%FBS (purchased from Santa Cruz Biotechnology, Inc, Santa Cruz, CA, USA) for further culture for 72 h. The experiment was performed in triplicate.

### 5-Ethynyl-2’-deoxyuridine (EdU) assay

Cell proliferation was detected using an EdU assay kit (C10310, RiboBio, Guangzhou, China) as per the manufacturer’s instructions. The cells were briefly exposed to EdU for a 2 h period, after which they were collected, fixed in cell fixation solution for approximately 20 min and then permeabilized with 0.5% TritonX-100 for 10 min. After PBS washing, the cells were treated with 1×Apollo for 30 min. The cells were then analyzed and photographed under a fluorescence microscope. Three fields were randomly selected followed by counting the EdU positive cells (red stained nuclei). EdU labeling rate (%) = number of positive cells/(number of positive cells + negative cells) × 100%. The experiment was conducted in triplicate.

### Transwell assay

The cells were then seeded into a Transwell (8 μm pore size) chamber with a density of 5 × 104 cells/ml (200 μL in each chamber). The exosomes were added into the upper chamber at a concentration of 50 μg/mL, with three duplicate wells set for each group. The Transwell chamber was cultured at 37°C in a humidified atmosphere comprised of 5% CO_2_ for 24 h. Twenty-four hours after treatment, the transwell chamber was washed twice with PBS, fixed using 5% glutaraldehyde, and stained with crystal violet dye solution (Beyotime Biotechnology Co., Shanghai, China) for 20 min, after which the cells on the internal surface of the chamber wiped off using a cotton ball. Crystal violet was eluted with 100 μL of 33% acetic acid, with an optical density (OD) at 570 nm examined using a microwell microplate reader. The cells were observed and photographed under an inverted fluorescent microscope (TE2000, Nikon, China). The experiment was performed in triplicate.

### Tube formation assay

Pre-cooled angiogenesis slide (81506, Ibidi GmbH, Martinsried, Germany) was coated with 10 μL Matrigel (354234, Shanghai Shanran Biotechnology Co., Ltd., Shanghai, China). The cells were collected and re-suspended in DMEM culture medium at a density of 2 × 105/mL and seeded into the Matrigel-coated slide, with three duplicated wells set accordingly. After the cells were incubated for 12 h, photographs were taken under a Leica inverted phase contrast microscope. A minimum of 3 visual fields were selected in each group with the number of capillary tubes calculated with image-pro plus (version 6.0). The experiment was conducted in triplicate.

### Dual-luciferase reporter assay

The target genes of miR-183-5p and binding sites were examined with the biological prediction website Target Scan. Dual-luciferase reporter assay was employed to clarify whether there exists a targeting relationship between FOXO1 and miR-183-5p. Next, a pGL3-FOXO1 Wt reporter plasmid was constructed, which contained 3’UTR of FOXO1 that contained the potential miR-183-5p binding sites. The mutant form, pGL3-FOXO1 Mut, in which the potential miR-183-5p binding sites were mutated, was then constructed. The two reporter plasmids were respectively co-transfected into the HEK293 cells with over-expressed miR-183-5p plasmid and pRL-TK (internal reference plasmid expressing Renllia luciferase). Then, 24 h following transfection, the cells were lysed in accordance with the instruction of the TransDetect Double-Luciferase Reporter Assay Kit (FR201-01, Beijing TransGen Biotech Co., Beijing, China). Luciferase activity was detected using a dual-luciferase® reporter assay system (E1910, Promega Corporation, Madison, WI, USA). The relative luciferase activity was determined based on the ratio of firefly luciferase to Renilla luciferase. The experiment was conducted in triplicate.

### Xenograft tumor in nude mice

A total of 20 Balb/c male nude mice (age: 4-6 weeks, weight: 16.66 g - 21.11 g; Shanghai Lab. Animal Research Center, Shanghai, China) were randomly selected and raised in a laminar flow clean room with a barrier system (SPF level) under controlled room temperature conditions of 24-26° C and relative humidity of 40%-60%. After the HT29 cells had been collected and resuspended at a density of 5 × 107/ml, 100 μL cell suspension was triturated again with 200 μL microsyringe, and injected in a cautious manner into the right scapular region of nude mice. Next, miR-183-5p antagomir (100 nM) was injected into the caudal vein of each nude mouse at regular intervals every other day. After 3 weeks, the mice were euthanized by intraperitoneal injection with 9% pentobarbital sodium (P3761, sigma, St. Louis, USA). Tumor was dissected and the short diameter (a) in addition to the length diameter (b) of the tumor was measured using a vernier caliper. The tumor volume was calculated based on the formula π (a2b)/6, while weight of the tumor weight was determined. Peripheral blood was collected from the nude mice in order to prepare serum. The serum exosomes were extracted in accordance with the aforementioned method with the objective of identifying the expression of miR-183-5p using qRT-PCR (with cel-miR-39 as internal reference). The tumor tissues were fixed by 10% formaldehyde, conventionally dehydrated, embedded by paraffin and sliced into 4 μm sections. Immunohistochemical detection of FOXO1 protein expression was then performed accordingly. The expression of VEGFA, FOXO1, VEGFAR-2, ANG2, PIGF, MMP-2 and MMP-9 in tumor tissues was detected by means of Western blot analysis.

### Immunohistochemistry

Fresh tissues were cut into 4 μm thick slices, which were subsequently subjected to immunostaining. Immunohistochemistry was performed in order to detect the expression of CD31 (#3528, 1:1600, Cell Signaling Technology, Boston, MA, USA) and FOXO1 (#2880, 1:100, Cell Signaling Technology, Boston, MA, USA). The expression of CD31 was analyzed under a microscope. CD31 was found to be predominately expressed in the cytoplasm/membrane of the endothelial cells which was represented by a brown color under a microscope. Microvessel density (MVD) was evaluated as per the immunohistochemical method described previously [38].

### Enzyme-linked immunosorbent assay (ELISA)

The peripheral blood was collected from the nude mice and the serum level of VEGFA was detected according to the instructions of the ELISA kit (A106111-48T, Fusheng Industrial, Shanghai, China).

### Statistical analysis

All experimental data were processed using SPSS 25.0 statistical software (IBM Corp., Armonk, NY, USA), while normal distribution and variance homogeneity evaluations were performed accordingly. Data were expressed as mean ± standard deviation in the event it conformed to normal distribution. Comparisons between two groups were conducted by means of t-test, while comparisons among multiple groups were assessed by one-way analysis of variance. Data at different time points were analyzed by repeated measurement analysis of variance. Statistically significance was indicated when the p value was < 0.05.

## Supplementary Material

Supplementary Figures
