# Supplementary material for: Exosomal miR-183-5p promotes angiogenesis in colorectal cancer by regulation of FOXO1
Source: Aging (Albany NY). 2020 May 3;12(9):8352–71. doi: 10.18632/aging.103145 (PMC7244076; doi:10.18632/aging.103145)
Supplement: Supplementary Figures [file aging-12-103145-s001..pdf]

SUPPLEMENTARY FIGURES

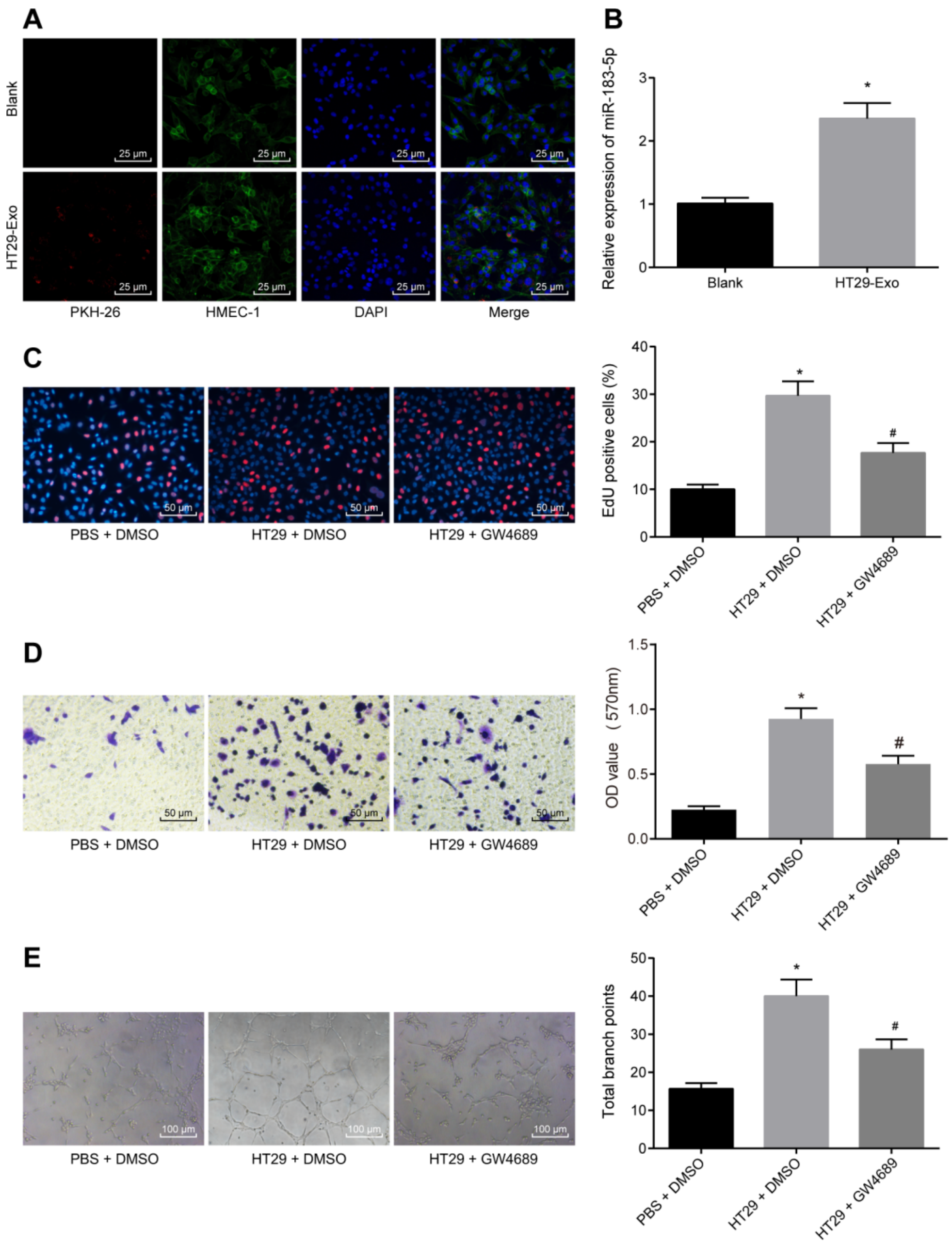

Supplementary Figure 1. HT29 cells-secreted exosomes promote proliferation, migration and tube formation abilities of HMEC-1 cells. (**A**) uptake of PKH-26-labeled HT29 cells-secreted exosome by HMEC-1 cells (Scale bar = 25 μm); (**B**) miR-183-5p expression in HMEC-1 cells co-cultured with HT29-exo detected via RT-qPCR, *p< 0.05 compared with the blank group; (**C**) HMEC-1 cell proliferation detected by EdU assay (Scale bar = 50 μm), *p < 0.05 compared with the PBS + DMSO group (HMEC-1 cells treated with PBS and DMSO), #p < 0.05 compared with the HT29 + DMSO group (HMEC-1 cells co-cultured with HT29 cells-secreted exosomes and DMSO); (**D**) HMEC-1 cell migration detected by Transwell assay(Scale bar = 50 μm), *p < 0.05 compared with the PBS + DMSO group, #p < 0.05 compared with the HT29 + DMSO group; (**E**) tube formation abilities of HMEC-1 cell detected by tube formation assay (Scale bar = 100 μm), *p< 0.05 compared with the PBS + DMSO group, #p < 0.05 compared with the HT29 + DMSO group. Measurement data were expressed as mean ± standard deviation; comparisons among multiple groups were assessed by one-way analysis of variance. Cell experiment was repeated three times.

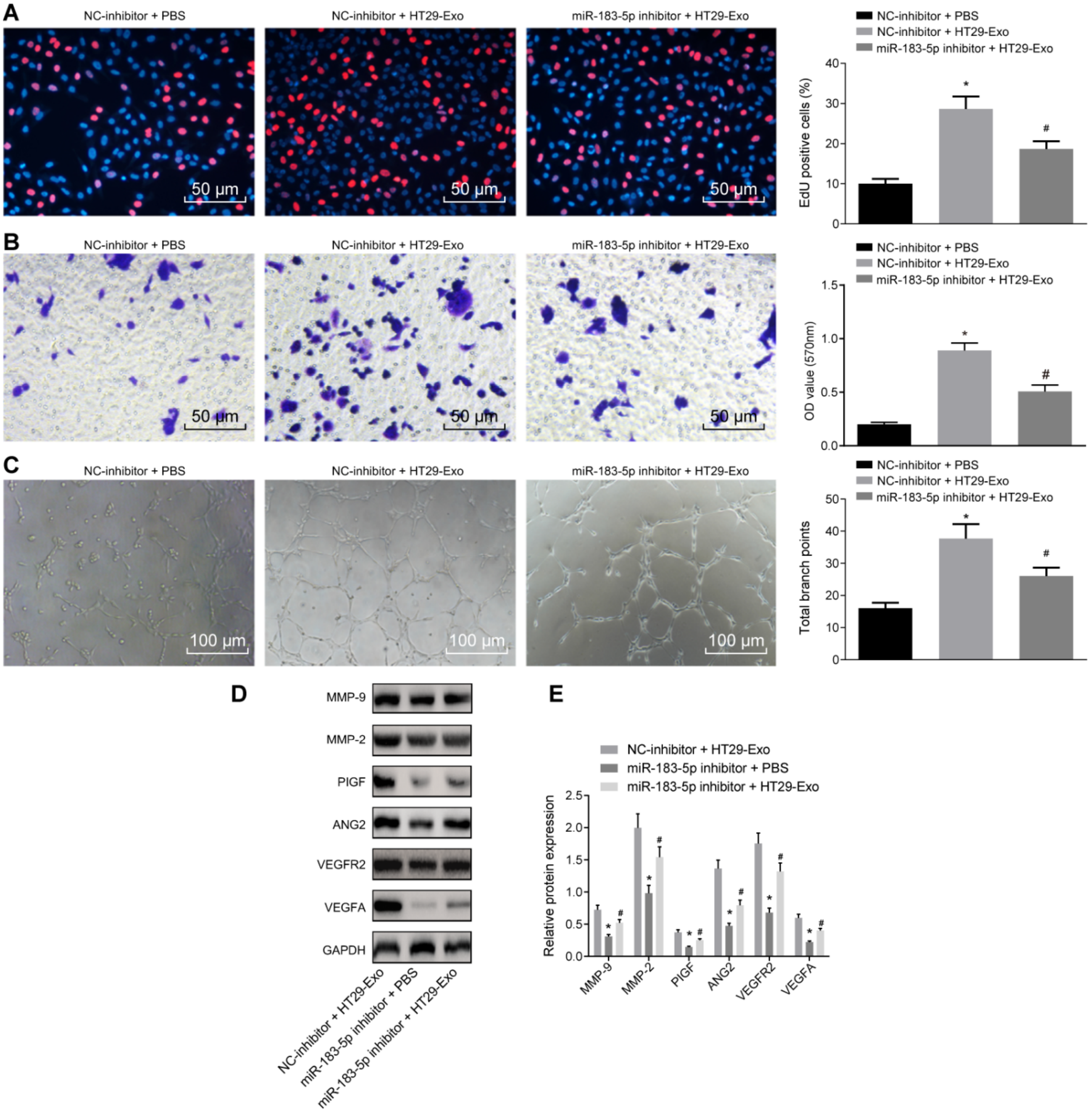

Supplementary Figure 2. Inhibition of miR-183-5p effectively reverses promoting effect of HT29-Exo on the facilitated proliferation, migration, tube formation and angiogenesis of HMEC-1 cells. (**A**) EdU assay was applied to detect proliferation of HMEC-1 cells after treatment of HT29-Exo and miR-183-5p inhibitor (Scale bar = 50 μm); (**B**) HMEC-1 cell migration was detected by Transwell assay after treatment of HT29-Exo and miR-183-5p inhibitor (Scale bar = 50 μm); (**C**) tube formation abilities of HMEC-1 cell were detected by tube formation assay after treatment with HT29-Exo and miR-183-5p inhibitor (Scale bar = 100 μm); (**D**–**E**) expression of angiogenesis-related proteins (VEGFA, VEGFAR2, ANG2, PIGF, MMP-2 and MMP-9) in HMEC-1 cells after treatment with HT29-Exo and miR-183-5p inhibitor detected by western blot analysis; *p < 0.05 compared with the NC-inhibitor + HT29-Exo group, #p < 0.05 compared with the miR-183-5p inhibitor + PBS group; Measurement data were presented as mean ± standard deviation; comparisons among multiple groups were assessed by one-way analysis of variance. Cell experiment was repeated three times.

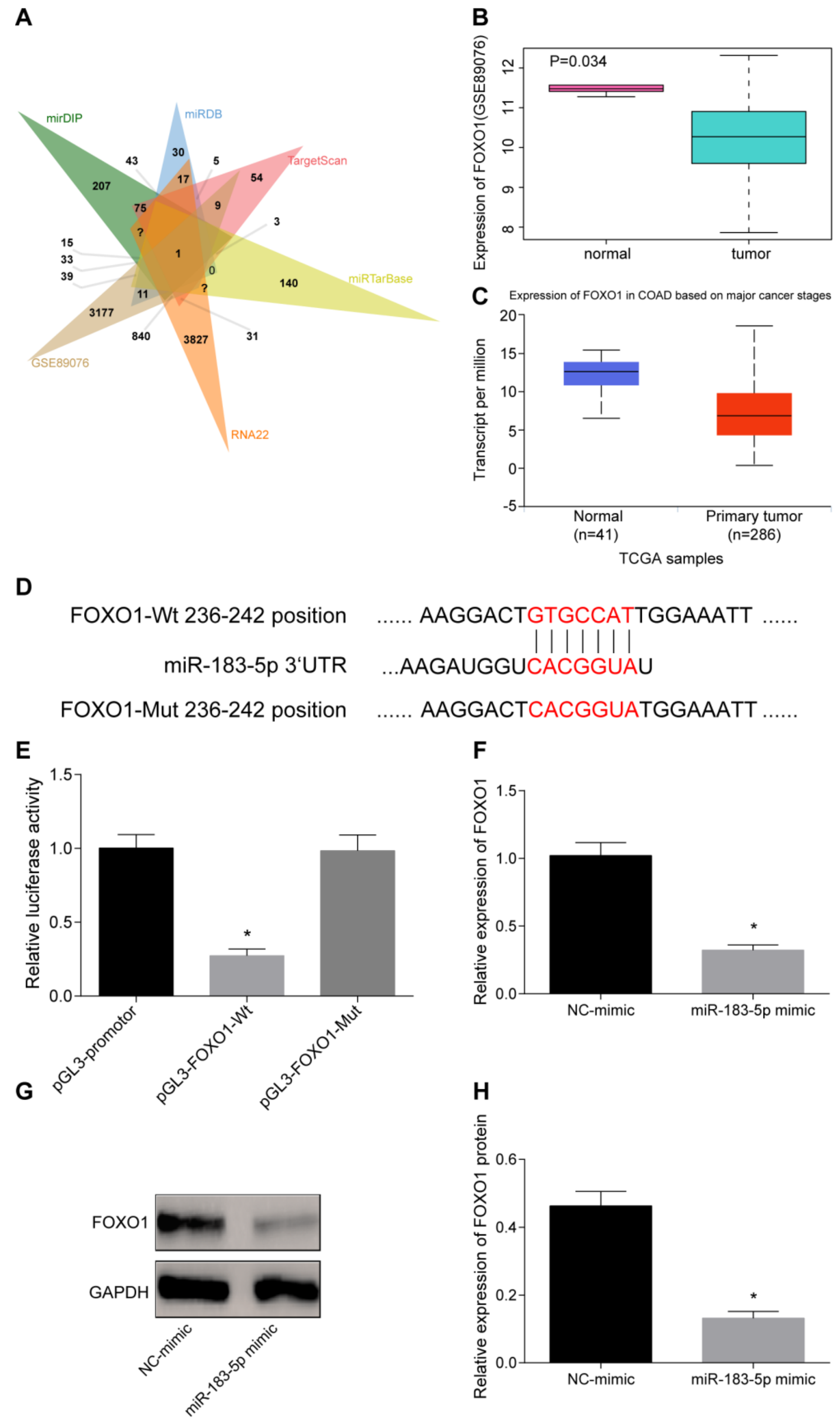

Supplementary Figure 3. FOXO1 is a potential target gene of miR-183-5p. (**A**) intersection of the target genes of miR-183-5p predicted by miRDB, mirDIP, TargetScan, miRTarBase, RNA22 and the differentially expressed genes screened from GSE89076, *$p < 0.05$ compared with pGL3-promotor; (**B**) expression of FOXO1 in GSE89076; (**C**) UALCAN database showed low expression of FOXO1 in CRC; (**D**) prediction of binding sites between miR-183-5p and FOXO1 by miRDB website; (**E**) verification of binding relationship between miR-183-5p and FOXO1 by dual luciferase reporter assay; (**F**) mRNA expression of FOXO1 detected by RT-qPCR; (**G** and **H**) protein expression of FOXO1 detected by western blot analysis; *$p < 0.05$ compared with the NC-mimic group; Measurement data were presented as mean ± standard deviation. Comparisons between two groups were conducted by means of independent *t*-test; comparisons among multiple groups were assessed by one-way analysis of variance. Cell experiment was repeated three times.

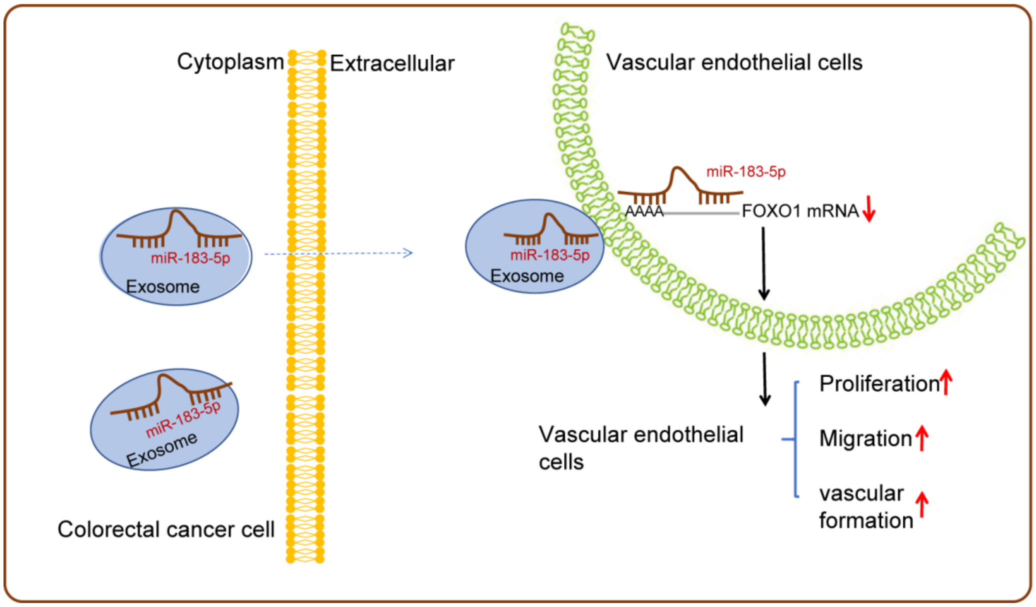

Supplementary Figure 4. The schematic representation illustrating functions of CRC-derived exosomes in vascular endothelial cells. Exosomes secreted from CRC cells exhibit high miR-183-5p expression, which can promote proliferation, migration and tube formation abilities of HMEC-1 cells by targeting and negatively regulating FOXO1.
